# Children and young people’s consultation rates for psychosocial problems between 2016 and 2021 in the Netherlands

**DOI:** 10.1080/13814788.2024.2357780

**Published:** 2024-06-04

**Authors:** Lukas B.M. Koet, Premysl Velek, Patrick J.E. Bindels, Arthur M. Bohnen, Evelien I.T. de Schepper, Heike Gerger

**Affiliations:** a Department of General Practice, Erasmus MC, University Medical Centre Rotterdam, Rotterdam, The Netherlands; b Department of Clinical Psychology, Open University, Heerlen, The Netherlands

**Keywords:** Child, young people, mental health, consultation rates, COVID-19

## Abstract

**Background:**

Worldwide, there are concerns about declining mental health of children and young people (CYP).

**Objectives:**

To examine trends in GP consultation rates for psychosocial problems and the impact of the COVID-19 pandemic.

**Methods:**

We performed a population-based cohort study using electronic GP records of CYP (0–24 years) living in the Rotterdam metropolitan area between 2016 and 2021. We calculated monthly consultation rates for psychosocial problems, stratified by age group and sex. We used negative binomial models to model the pre-COVID-19 trend, and estimate expected rates post-COVID-19 onset. We modelled the effect of COVID-19 infection rate and school closure on consultation rates per sex and age group.

**Results:**

The cohort increased from 64801 to 92093 CYP between January 2016 and December 2021. Median age was 12.5 years and 49.3% was female. Monthly consultation rates increased from 2,443 to 4,542 consultations per 100,000 patient months over the six years. This trend (RR 1.009, 95%CI 1.008–1.011) started well before the COVID-19 pandemic. Consultation rates of adolescent girls and young women increased most strongly. Between March and May 2020, there was a temporary reduction in consultation rates, whereupon these returned to expected levels. COVID-19 infection rate and school closures showed small but significant associations with consultation rates for psychosocial problems but this did not affect the overall trend. Although consultation rates for psychosocial problems increased, this increment was stable over the entire study period.

**Conclusion:**

The COVID-19 pandemic did not significantly increase consultation rates for psychosocial problems in CYP. The consultation rates increased, especially in adolescent girls and young women.

## KEY MESSAGES

CYP’ consultation rates for psychosocial problems within general practice almost doubled between 2016 and 2021 in the Rotterdam metropolitan area, the Netherlands.

This increase was steady over time and was not affected by the COVID-19 pandemic.

Consultation rates in general practice for psychosocial problems increased most strongly in adolescent girls and young women

## Introduction

There is much concern about the adverse effects of the COVID-19 pandemic and the associated public health measures on the mental health of children and young people (CYP) [1]. According to a meta-analysis, the global prevalence of depression and anxiety symptoms in children and adolescents doubled during the first year of the pandemic [2]. Also in the Netherlands, CYP reported more mental health problems compared to pre-pandemic periods [3].

Mental health problems in CYP significantly impair quality of life and have long-term disabling effects [4, 5]. To mitigate those effects, CYP should receive appropriate treatment [6]. During the COVID-19 pandemic, temporary closures of many mental health services reduced access to appropriate care [7,8]. Indeed, research shows that the use of mental health services by CYP decreased during the early phase of the pandemic, highlighting potential delays or unmet needs for treatment [9].

In the Netherlands, CYP presenting with psychosocial problems can be managed within general practice, sometimes by a specialised practice nurse or referred to specialised care [10,11]. Accordingly, Dutch GPs play an important role in identifying, managing and referring CYP with psychosocial problems [12]. The COVID-19 pandemic may have increased the importance of GPs in managing psychosocial problems of CYP (e.g. because of restricted access to specialised mental health services) [13].

Currently, information on the trends of GP consultations by CYP for psychosocial problems particularly during the COVID-19 pandemic is scarce. Also, it is unclear to what extent consultation rates were affected by the severity of the pandemic and the associated public health measures.

In this study, we investigated trends in monthly GP consultation rates for psychosocial problems in CYP between 2016 and 2021 and the impact of the COVID-19 pandemic on consultation rates by sex and age.

## Methods

### Study design and population

We used electronic records of children and young people (CYP) aged 0–24 years registered in general practices participating in the Rijnmond Primary Care Database (RPCD) between January 1^st^, 2016 and December 31^st^, 2021. The RPCD is a region-specific derivative of the Integrated Primary Care Information (IPCI) database, covering the Rotterdam metropolitan area [14]. The RPCD currently consists of more than 600,000 patients. It contains pseudonymised routinely collected medical data of general practice patients, including all written GP notes, diagnostic codes, referrals, laboratory findings, GP prescriptions, and specialists’ letters. In the Netherlands, GPs use the International Classification for Primary Care 1 (ICPC-1) to code the reasons for consultations [15].

### Calculation of consultation rates

We extracted (1) all GP consultations and (2) all patients with valid database information for each month. We calculated the monthly consultation rates for psychosocial and non-psychosocial problems (GP consultations per 100,000 patient months) for the complete cohort and per age group and sex. We defined psychosocial consultations as any contact between a GP and a patient (in person, by phone, or online) coded with any P-code (psychological problems, e.g. anxiety disorder), any Z-Code (social problems, e.g. relationship problems with parents/family), R98 (hyperventilation) or T06 (eating disorder). Supplementary Material Table 1 shows an overview of psychosocial ICPC codes, the most common psychosocial problems included ADHD, anxiety problems, depressive problems and learning problems. We classified other contacts as non-psychosocial consultations. We defined four age groups: young children (0–6 years), primary school children (7–12 years), high-school adolescents (13–17 years), and young adults (18–24 years).

We also calculated non-psychosocial consultation rates for the complete cohort to assess whether the trend differed between non-psychosocial and psychosocial consultations.

### Data-analysis

We fitted negative binomial models to assess our research questions (R version 4.0.0, MASS package) [16]. In our models we adjusted for monthly seasonality and a linear trend over time. For selection of our models we used backward stepwise selection and reduced models one-by-one variable using F and likelihood-ratio tests with a cut-off value (*p* < 0.05; Supplementary Material Table 2). Residual plots were used to validate the models’ assumptions. In Model 1 we used data from January 2016 until February 2020 to estimate the expected consultation rates for psychosocial problems for the pandemic period (March 2020 to December 2021). We used Model 1 to calculate the expected number of consultations for the first COVID-19 wave (March-May 2020) and the complete COVID-19 period (March 2020–December 2021) by summing up the expected monthly rates with their 95%CI and compared these with the observed numbers.

We repeated this analysis for non-psychosocial problems (Model 2) to allow for comparisons of trends between psychosocial and non-psychosocial problems (i.e. to critically evaluate whether changes in consultation rates for psychosocial problems are different from overall changes in consultation rates over time). Next, we used the complete study period to investigate in which manner COVID-19 impacted consultation rates (Model 3) and whether this differed between age groups and sexes (Model 4). For this, we used ‘OurWorldInData.org,’ which contains daily statistics on the COVID-19 pandemic for every country [17]. We considered five indicators of COVID-19 pandemic severity to be of possible relevance: ‘COVID-19 infection rate,’ ‘COVID-19 death rate,’ ‘COVID-19 hospitalisation rate,‘ ‘COVID-19-stringency index,’ ‘school-closure index’ [17, 18]. School closure is expressed on four levels, ranging from no measures to closing off all school levels [18]. These indices, averaged per month, were tested as covariates. There was multicollinearity between these indicators, with high values on the variance inflation factor (VIF > 10) and significant coefficient changes when removing individual coefficients. We selected the ‘school-closure index’ and ‘COVID-19 infection rate’ as predictors in the final models as they were the most relevant predictors showing no multicollinearity. In Model 3 we investigated the overall impact of ‘school-closure index’ and ‘COVID-19 infections’ on CYP’ consultation rates. In Model 4, we tested for heterogeneity in the effect of ‘school-closure index’ and ‘COVID-19 infections’ in different age groups and sexes by testing for interactions between these variables (Supplementary Material Table 2).

### Sensitivity analyses

We repeated the previously described analyses using the total number of unique patients consulting per month instead of the total number of consultations per month (i.e. if a patient had two consultations in one month this was counted as one unique patient).

### Ethics and data availability

The study was approved by the Governance Board of the RPCD (project number 2020.012). All patient data was pseudonymised. Therefore, by Dutch law, no patient consent is required. We followed the RECORD guidelines for studies conducted using routinely collected health data [19]. Due to legal constraints, data is not publicly available and access requires approval from the Governance Board of RPCD.

## Results

### Cohort characteristics

The number of CYP (0–24 years) in our database, which consists of practices in the Rotterdam metropolitan area, increased from 64801 patients in January 2016 to 92093 patients in December 2021 due to more general practices providing data to the RPCD. The median age was 12.5 years (IQR 6.5–18.5), and 49.3% were female. Approximately 20% of CYP lived in a socially deprived area [20]. These cohort characteristics remained stable over time (Table 1).

**Table 1.** Study population demographics.

Year	N Population size	Sex (% Female)	Median age (IQR)	Deprivation status (% registered in deprived postal code)
2016	64801	49.3%	12.5 (6.5–18.5)	19.2%
2017	74788	49.2%	13 (6.5–19)	20.6%
2018	80501	49.1%	13 (6.5–19)	20.0%
2019	79210	49.1%	13 (6.5–19)	20.5%
2020	82802	49.1%	13 (6.5–19)	20.4%
2021	96635	49.1%	12.5 (6.5–18.5)	21.9%

The RPCD is a dynamic cohort and the study population differed per month. Numbers represent the month January of each year.

### Consultation rates per age and sex categories

**Figure 1.**
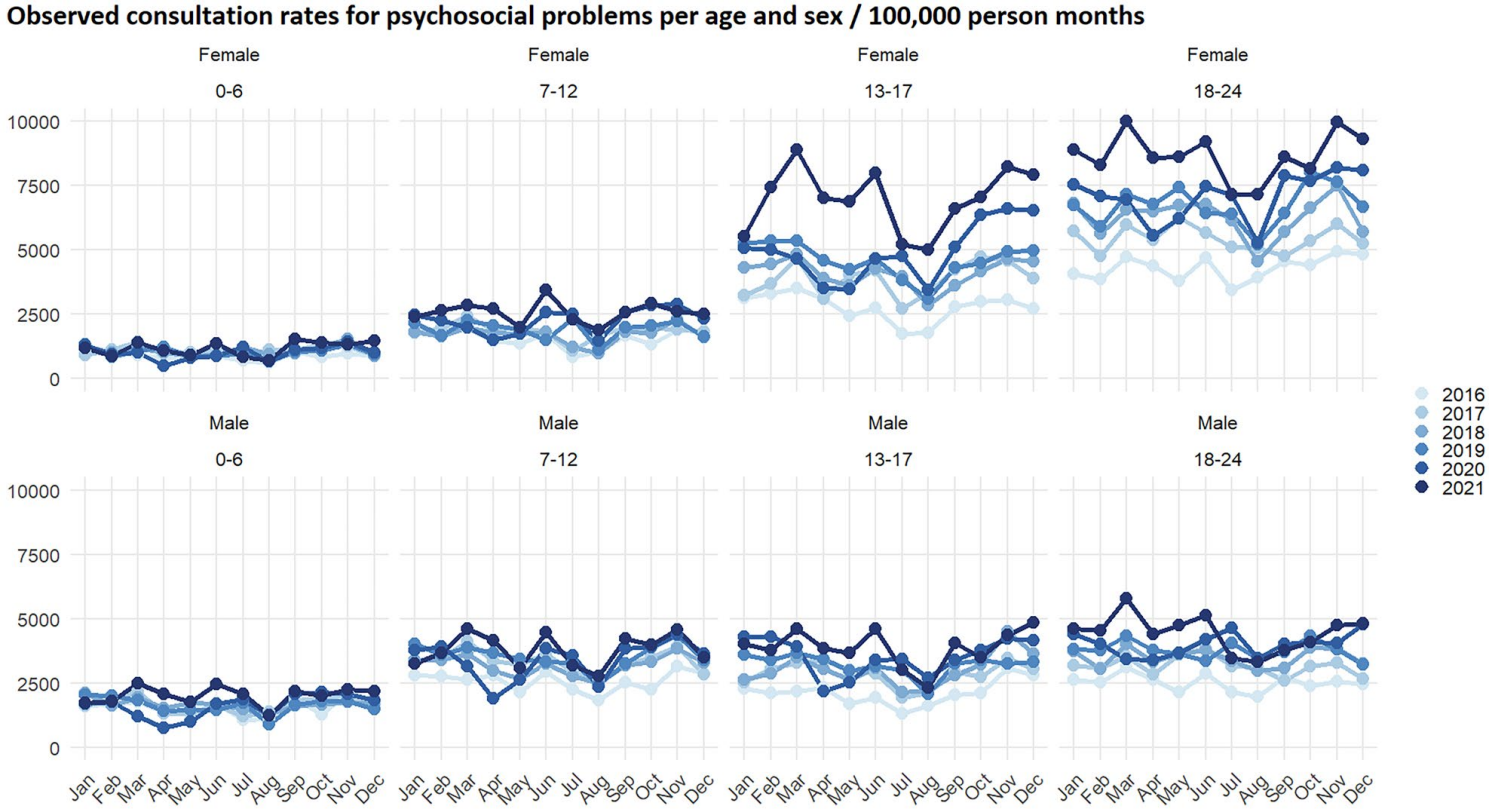
Consultation rates per age and sex categories over time.

By visually inspecting the observed consultation rates for psychosocial problems differences between age groups and sexes can be seen (Figure 1, Supplementary Material Figure 1). Consultation rates for psychosocial problems were lowest in young children (0–6 years), while consultation rates for psychosocial problems were highest in girls (13–17 years) and young women (18–24 years).

### Time trend of consultation rates

**Figure 2.**
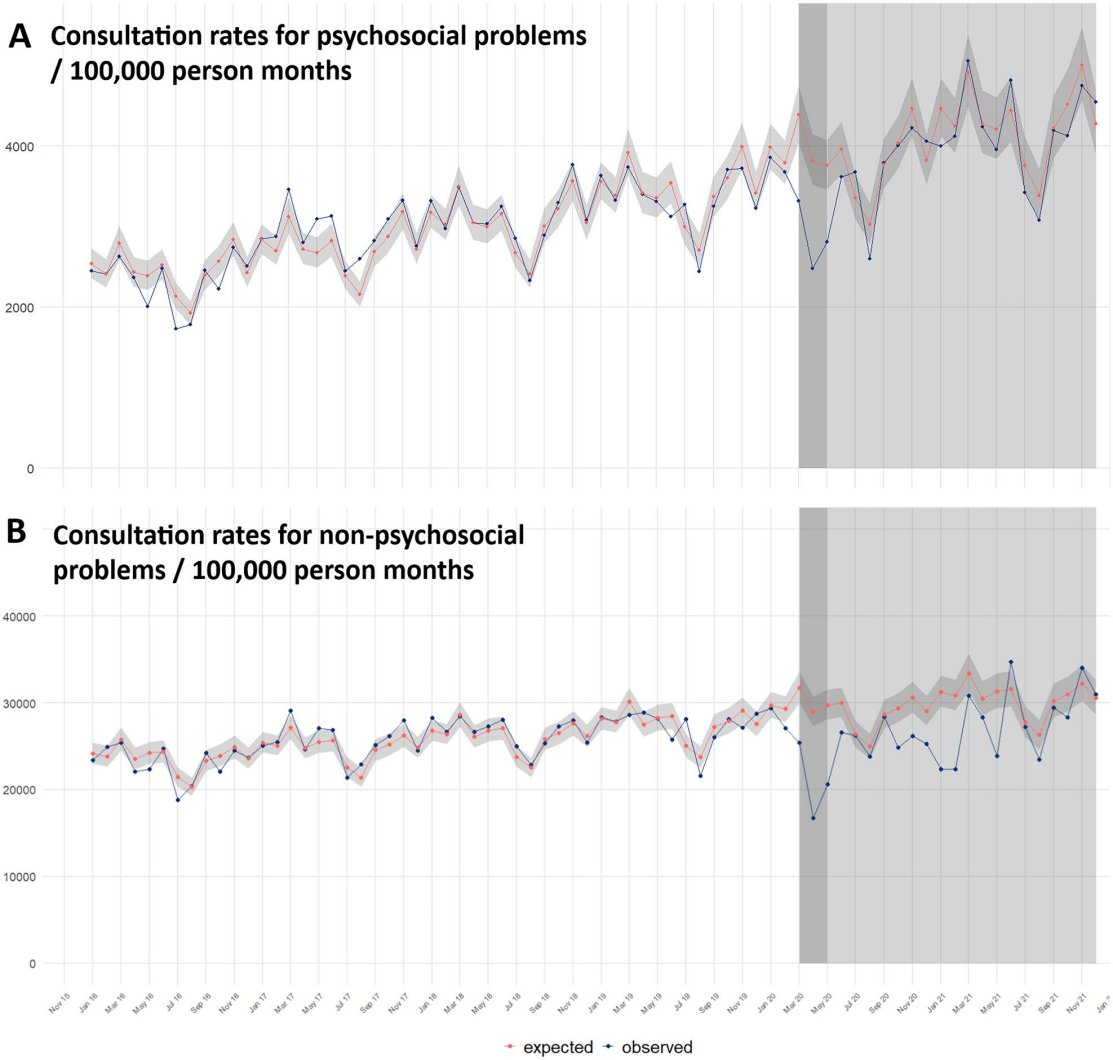
Expected and observed consultation rates over time. Figure 2a shows the observed monthly psychosocial consultation rates and the expected rates based on Model 1. Figure 2b shows the observed non-psychosocial rates and the expected rates based on Model 2. The shaded area indicates the pandemic period with the first wave in dark grey.

The monthly consultation rate for psychosocial problems increased strongly from 2,443 consultations per 100,000 patient months in January 2016 to 4,542 consultations per 100,000 patient months in December 2021. Visual inspection showed a linearly increasing trend with reasonable seasonal variation (e.g. lower consultation rates during summer holidays; Figure 2a). Consultation rates for non-psychosocial problems showed a much smaller increase over time (Figure 2b). This is reflected in Model 1 and 2, in which consultation rates for psychosocial problems show a stronger increase in the pre-pandemic period than those for non-psychosocial problems (relative rate (RR) 1.009 [95%CI 1.008–1.011] vs 1.004 [95%CI 1.003–1.005] per month, Supplementary Material Table 3).

### Impact of the COVID-19 pandemic on consultation rates: Observed vs expected consultations

Model 1 and 2 estimated consultation rates for psychosocial and non-psychosocial problems during the COVID-19 pandemic based on pre-pandemic data and aimed to compare expected and observed rates. Using visual inspection, we saw a decrease in consultation rates for both psychosocial and non-psychosocial problems during the first COVID-19 lockdown in March-May 2020 compared to expected rates (Figure 2). Hereafter, the rates for psychosocial problems returned to expected levels without clear indications of significant changes in subsequent lockdowns.

A total of 7,382 psychosocial consultations occurred during the first COVID-19 lockdown (March-May 2020). Based on Model 1 the number of observed consultations was 28.1% significantly lower than expected (expected: 10,262 consultations 95%CI 9,459–11,134; Supplementary Material Table 4). In the complete COVID-19 period (March 2020–December 2021) a total of 82,941 consultations took place which was 5.4% non-significantly lower than expected (87,637 95CI% 80,398–94,876) based on Model 1.

### Impact of COVID-19 infection rate and school closures on observed consultation rates

In Model 3 we tested whether COVID-19 infection rates and school closures affected observed consultation rates. In Model 3 seasonality, trend over time, ‘COVID-19 infection rate’, and ‘school-closure index’ were all significantly associated with observed consultation rates for psychosocial problems (Table 2). Complete school closure was associated with an overall reduction in observed consultation rates with a RR of 0.786 (95%CI 0.706–0.875) when compared with periods with no school closure.

**Table 2.** Regression table of model 3 estimating the effect of COVID-19 indices on consultation rates.

Variables	Estimate of relative rate (RR) (95% CI)	*p* Value
Trend over time (RR per subsequent month)	1.010 (1.008–1.011)	*p* < 0.001
School closure^a^	0.786 (0.706–0.875)	*p* < 0.001
COVID-19 infection rate^b^	1.010 (1.002–1.018)	*p* = 0.010

Only significant variables were kept in the final model. We adjusted for effects of seasonality. For the complete model including seasonality variables see Supplementary Material Table 3.

^a^School closure is a levelled index, transformed to a scale from 0 to 1, averaged per month. 0 represents a complete month without restrictions, 1 represents a month with complete school closure.

^b^COVID-19 infection rate represents the national number of daily new cases per 1000 inhabitants, averaged per month.

A higher COVID-19 infection rate was associated with a slight increase in observed consultation rates for psychosocial problems. An increase in the average daily number of new COVID-19 infections 1 per 1,000 inhabitants was associated with an increase in observed consultation rates with an RR of 1.010 (95%CI 1.002–1.018).

In Model 4 we tested whether the influence of COVID-19 infection rate and school closures on GP consultation rates for psychosocial problems differed per age group and sex (Table 3). The effect of school closure differed per age group. In children (0–6 years), complete school closure was associated with the most significant decrease in the consultation rate (RR 0.563, 95%CI 0.459–0.692). School closure did not have a different effect on consultation rates between males and females. Additionally, we did not find a significant difference in the effect of the COVID-19 infection rate on consultation rates between different age groups or between sexes.

**Table 3.** Regression table of model 4 estimating the effect of COVID-19 indices in different age categories.

Variable	Estimate of relative rate (RR) (95% CI)	*p* Value
Trend over time (RR per subsequent month)	1.009 (1.007–1.010)	*p* < 0.001
Age 7–12	1.862 (1.725–2.010)	*p* < 0.001
Age 13–17	2.513 (2.329–2.712)	*p* < 0.001
Age 18–24	3.361 (3.116–3.626)	*p* < 0.001
School closure^a^ in: Age 0–6 Age 7–12 Age 13–17 Age 18–24	0.563 (0.459–0.692) 0.693 (0.565–0.848) 0.951 (0.777–1.163) 0.891 (0.729–1.089)	*p* < 0.001^c^
COVID-19 infection rate^b^	1.013 (1.003–1.023)	*p* = 0.009

The reference group is the consultation rate for children aged 0–6. Only significant variables and interactions were kept in the final model. Sex was no significant predictor. The effect of the different months (seasonality) is not shown in this table (Supplementary Material Table 5 for complete model). ^a^School closure is a levelled index, transformed to a scale from 0 to 1, averaged per month. 0 represents a full month without restrictions, 1 represents a month with complete school closure. ^b^COVID-19 infection rate represents the national number of daily new cases per 1000 inhabitants, averaged per month. ^c^There was a significant interaction between school closure and the different age categories, the effect is shown per age category, the presented *p* value derived from likelihood-ratio test of the interaction components.

### Sensitivity analyses

Sensitivity analyses showed similar results to our primary analyses (Supplementary Material Figures 3 and 4, Supplementary Material Tables 6 and 7). Our analyses using unique patients showed that the number of CYP consulting the GP with a psychosocial problem increased over time (RR 1.007, 95%CI 1.006–1.008 per month), indicating that the overall increase in consultation rates over time can be explained by both more CYP contacting the GP and more consultations per patient.

Our main analyses showed that consultation rates for psychosocial problems increased, especially in adolescent girls (13–17 years) and young women (18–24 years). We, therefore, performed two post-hoc exploratory subgroup analyses for these categories (Supplementary Material Tables 2 and 8). The subgroup analyses with adolescent girls (13–17 years) and young women (18–24 years) were in line with our main analyses, except for a small, temporary increase in the consultation rates for adolescent girls between March–June 2021 (Supplementary Material Figure 5).

## Discussion

### Main findings

In our study, monthly consultation rates for psychosocial problems have been increasing strongly since 2016, especially among adolescent girls and young women. Consultation rates for both psychosocial and non-psychosocial problems dropped during the first COVID-19 wave. Hereafter consultation rates for psychosocial problems continued increasing following the pattern that was present before the start of the COVID-19 pandemic. Consultation rates for non-psychosocial problems remained lower than expected until the summer of 2021. Overall, the COVID-19 pandemic did not increase GP consultation rates in CYP for psychosocial problems.

### Strengths and limitations

Our study has several strengths. First, we used the RPCD, a large population-based dynamic cohort representative of the Rotterdam metropolitan area. Contrary to many other studies on the effects of the COVID-19 pandemic using cross-sectional samples, we used data from six consecutive years, covering the peak of the COVID-19 pandemic in 2020 and 2021. This allowed for considering the trend of increasing health-seeking behaviour that had started before the pandemic. Second, we applied several sensitivity analyses, which yielded very similar results, confirming the validity of our findings. Third, we used information from ‘OurWorldinData.org,’ to measure the influence of the COVID-19 pandemic on consultation rates [17]. This database offers open access to statistical data on the COVID-19 pandemic for every country worldwide, making it possible to repeat our analysis in different settings. Fourth, we compared the overall trend for consultations for psychosocial problems with the overall trend for consultations for non-psychosocial problems to check whether the observed increases are to be seen as a general trend for more GP consultations by CYP during the study period.

This study also has limitations. First, in electronic healthcare databases, such as the RPCD coding by healthcare professionals may be imprecise. For instance, the ICPC coding systems allow for coding anxiety-related problems as P01 feeling anxious/stressed or as P74 anxiety disorder. However, as we included a broad range of psychosocial problems without differentiating between the individual problem areas, we do not think these imprecisions significantly affected our results. Second, we investigated overall consultation rates related to psychosocial problems without analysing specific mental health problems separately. Third, due to database properties, we could not differentiate between different types of consultation (e.g. by phone or in-person). Fourth, the RPCD is restricted to one metropolitan region in the Netherlands. Our findings might not necessarily generalise to more rural areas or other countries. Fifth, we used a dynamic cohort. Therefore the study population may change with general practices, such as joining or leaving the RPCD. However, in our cohort, the distribution of relevant patient characteristics remained stable. Finally, it is essential to realise that our study investigated help-seeking for psychosocial problems in general practice and not the occurrence of psychosocial problems per se. Our results should therefore be interpreted jointly with those of other types of studies (e.g. prevalence studies).

### Comparison with existing literature

A limited number of studies investigated long-term changes in primary care consultations for psychosocial problems in CYP during the COVID-19 pandemic [21–24]. Most of these studies showed an initial sharp decrease in consultation rates, followed by increases later in the pandemic to rates above pre-pandemic levels [21, 22, 24]. Additionally, the increased consultation rates for psychosocial problems seemed most prominent in girls, especially those in adolescence [21, 22, 24]. Our findings confirm initially decreased consultation rates during the first pandemic wave, which increased afterwards. Importantly, we show that the increasing trend in consultation rates began well before the COVID-19 pandemic. This finding complements previous research describing a general increase in clinical diagnoses for psychiatric disorders in CYP over decades up to 2015 [25], as well as increases in medical outpatient visits resulting in mental health diagnoses between 1995 and 2012 [26]. COVID-19 lockdowns and school closures have been linked to decreased mental health in CYP [27, 28]. Therefore, one can argue that more CYP would have sought help during forced school closure. Instead school closure was associated with decreased GP consultation rates in our study. Our finding that high COVID-19 infection rate showed a weak association with higher consultation rates for psychosocial problems suggests that the severity of the pandemic (i.e. quick viral spread) led to a tendency for more help-seeking by CYP. This association might be explained by raised psychological distress and anxiety about the consequences of infection (possibly mediated by extensive media attention to the COVID-19 virus) [29].

### Implications

Our analyses focused on consultation rates of CYP for psychosocial problems and showed not only an increase, especially in adolescent girls and young women but also an increasing gap between the sexes. These findings are in line with recent reports on the occurrence of psychosocial problems in CYP, which similarly show a higher prevalence of psychosocial problems in adolescent girls and young women and an increasing gap between sex-related prevalence to the disadvantaged girls [3, 30, 31], However, it is known that teenage boys and young men are less likely to seek help for psychological problems compared with their female peers [32], putting them potentially at risk of under detection. Future research should address these two possible explanations for the observed differences in consultation rates between males and females in our study. Additionally, it is crucial to investigate whether the observed increases in consultation rates, seen mainly in adolescent girls and young women, are the same across different psychosocial problems.

## Conclusion

Consultation rates for psychosocial problems in CYP within general practices in the Rotterdam metropolitan area have increased strongly between 2016 and 2021. The most significant increase in consultation rates was observed among adolescent girls and young women. Importantly, this trend started well before the COVID-19 pandemic. In our study, consultation rates for psychosocial problems in CYP did not increase due to the COVID-19 pandemic.

## Supplementary Material

Supplemental Material

Supplemental Material

## Data Availability

Due to legal constraints, data is not publicly available and access to the data requires approval from the Governance Board of RPCD.
