# Correction

**DOI:** 10.1080/13814788.2025.2570007

**Published:** 2025-10-30

**Article title:** Children and young people’s consultation rates for psychosocial problems between 2016 and 2021 in the Netherlands

**Authors:** Koet, L. B. M., Velek, P., Bindels, P. J. E., Bohnen, A. M., de Schepper, E. I. T., & Gerger, H.

**Journal:** *European Journal of General Practice*

**Bibliometrics:** Volume 30, Number 1, pages

**DOI:** https://doi.org/10.1080/13814788.2024.2357780

When the above article was first published, the research project number mentioned in the ‘Ethics and data availability’ paragraph on p. 3 was incorrectly listed as 2020.012. The correct project number is 2022.032. This has been amended in the online version.

