# Supplementary material for: Children and young people’s consultation rates for psychosocial problems between 2016 and 2021 in the Netherlands
Source: Eur J Gen Pract. 2024 Jun 4;30(1):2357780. doi: 10.1080/13814788.2024.2357780 (PMC11151797; doi:10.1080/13814788.2024.2357780)
Supplement: Supplemental Material [file IGEN_A_2357780_SM6849.docx]

**Title: Children and Young People’s consultation rates for psychosocial problems between 2016 and 2021**

**Authors:** Lukas B.M. Koet, MD^1^, Premysl Velek MSc^1^, Patrick J.E. Bindels, PhD^1^, Arthur M. Bohnen PhD^1^, Evelien I.T. de Schepper PhD^1^, Heike Gerger PhD^1, 2^

**Author affiliation:** 1) Department of General Practice, Erasmus MC, University Medical Centre Rotterdam, Rotterdam, the Netherlands. 2) Department of Clinical Psychology, Open University, Heerlen, the Netherlands

**Adress correspondence to:** Lukas Koet, Department of General Practice, Erasmus MC, University Medical Centre Rotterdam, Doctor Molewaterplein 50, 3000 CA Rotterdam, the Netherlands. E-mail: l.koet@erasmusmc.nl Telephone: +31 (0)10 703 0 004

eTable1. Overview of the type of psychosocial problems (ICPC codes) as percentage of the total of psychosocial problems

| **ICPC code** | **Description** | **% of total** |
| --- | --- | --- |
| P21 | ADHD | 11.73 |
| P24 | Specific learning problems | 9.55 |
| P74 | Anxiety disorders | 7.54 |
| P03 | Feeling depressed | 6.78 |
| P22 | Other worries about child’s behavior | 5.97 |
| P99 | Other psychiatric problem (including autism spectrum) | 5.9 |
| P76 | Depressive disorders | 5.76 |
| P01 | Feeling anxious/nervous/stressed | 5.37 |
| P29 | Other psychiatric symptoms/complains | 4.22 |
| P02 | Crisis/transient stress reaction (including PTSD) | 4.03 |
| P06 | Sleeping problems | 2.53 |
| P20 | Problems with memory/concentration/orientation | 2.04 |
| R98 | Hyperventilation | 1.63 |
| P19 | Substance (eg, drugs) abuse | 1.49 |
| P80 | Personality disorder | 1.38 |
| Z20 | Relationship problems with parents/family | 1.38 |
| P23 | Other worries about adolescent’s behavior | 1.35 |
| P12 | Enuresis | 1.32 |
| Z25 | Problem due to violence (or sexual assault) | 1.31 |
| P04 | Irritable/angry behavior | 1.28 |
| T06 | Eating disorder | 1.18 |
| Z23 | Loss/death of parents/family | 1.12 |
| P78 | Emotional exhaustion | 1.12 |
| P85 | Mental retardation | 1.1 |
| P11 | Eating problem of child | 0.95 |
| P79 | Other neurosis (including fobia and OCD) | 0.88 |
| Z21 | Problem due to behavior (including substance abuse) of parents/family | 0.85 |
| P17 | Tobacco abuse | 0.81 |
| P10 | Stammering/stuttering/tics | 0.75 |
| Z29 | Other social problem (including burnout) | 0.71 |
| Z16 | Relationship problem with child (including maltreatment/sexual abuse of child) | 0.71 |
| P09 | Concerns about sexual preferences (including gender dysphoria) | 0.69 |
| Z22 | Problem due to disease of parent/family | 0.66 |
| P77 | Suicide attempt (including suicid) | 0.63 |
| P98 | Other/non-specific psychosis | 0.60 |
| Z12 | Relationship problems with partner | 0.50 |

*ICPC codes representing less than 0.5% of total are not shown. These codes together represent less <5% of total number of psychosocial ICPC codes. **These numbers represent counts all ICPC codes coded by GPs. On average 103.8 psychosocial ICPC codes were given per 100 consultations. Thus, approximately 1 in 40 consultation was coded with two or more psychosocial ICPC codes.

eTable 2. Selection procedure for models

| Variables | Outcomes:  N: total number of psychosocial consultations  N2: total number of psychosocial consultations  Independent variables:  Month: categorical variable with 12 levels for every month  Trend: linear trend ranging for every month during the study period ranging from 1 (January 2016) to 72 (December 2021)  Sex: factorial variable with 2 levels (female 0; male 1)  Age category: factorial variable with 4 levels (0-6 years, 7-12 years, 13-17 years, 18-24 years)  Covid-19 New Cases: this variable was derived using information from OurWorldinData.org (initiative Oxford University), which presents daily COVID-19 statistics for 219 countries. Per month we averaged the daily number of new COVID-19 infections per 1000 inhabitants. This variable was used to investigate the influence of severity of the pandemic (ie, the viral spread)  School closure: this variable was derived using information from OurWorldinData.org (initiative Oxford University), which presents daily COVID-19 statistics for 219 countries. On OurWorldinData.org school closure is presented as variable with four levels (0 - no measures  1 - recommend closing or all schools open with alterations resulting in significant differences compared to non-Covid-19 operations  2 - require closing (only some levels or categories, eg just high school, or just public schools) 3 - require closing all levels). These were averaged per month and transformed to a scale from 0 to 1. 0 represents a complete month without restrictions, 1 represents a month with complete school closure.  Offsets:  Totals: For every month the number of person months (CYP<25 years)  Totals_category: For every month the number of person months (CYP<25 years) per age and sex  For the variables School closure and Covid-19 New Cases: [covid-19-data/public/data at master · owid/covid-19-data · GitHub](https://github.com/owid/covid-19-data/tree/master/public/data) |
| --- | --- |
| Model selection | For each model we started with a full model (see below for full model). We removed variables one-by-one, starting with removal of the least significant variable in the model. For every reduction we compared the reduced model with the non-reduced model using likelihood ratio test (LRT). When a variable LRT was significant (P value < 0.05) it was kept into the model, if not it was removed. This was continued until all remaining variables were significant. In case of model with interaction, we started reducing the model by removing the interactions. |
| Model 1 | Model 1_Full <- glm.nb( N ~ Trend + Month + offset(log(Totals)), data = dfm) dfm: data from January 2016 to February 2020  All tested variables were significant predictors. Full model = Final model |
| Model 2 | Model 2_Full <- glm.nb( N2 ~ Trend + Month + offset(log(Totals)), data = dfm) dfm: data from January 2016 to February 2020.  All tested variables were significant predictors. Full model = Final model |
| Model 3 | Model 3_Full <- glm.nb( N ~ Trend + Month + Covid-19 New Cases + School closure + offset(log(Totals)), data = dfm) dfm: data from January 2016 to December 2021.  All tested variables were significant predictors. Full model = Final model |
| Model 4 | Model 4_Full <- glm.nb( N ~ Trend + Month + Age category + Sex + Covid-19 New Cases + School closure + (Age category: Covid-19 New Cases) + (Sex :Covid-19 New Cases) + (Age category: School closure) + (Sex : School closure) + offset(log(Totals_category)), data = dfm) dfm: data from January 2016 to December 2021.  We first reduced the model by removing the interaction between Age category and Covid-19 New Cases. Secondly, we removed interaction between  Sex and Covid-19 New Cases. Thirdly, we removed the interaction between Sex and School Closure. Fourthly, we tried to remove the interaction between Age category and School closure. However, this decreased the fit of the model significantly and this interaction remained in the model. Finally, we removed sex because this did not improve the fit.  Model 4_Final <- glm.nb( N ~ Trend + Month + Age category + Sex + Covid-19 New Cases + School closure + (Age category: School closure) + offset(Totals_category), data = dfm  We did a post-hoc analysis checking whether the Covid-19 indices estimates changed when adding Sex and (Sex: Age category) to the model. This did not affect the COVID-19 indices estimates |
| Sensitivity analysis: | Unique patients: We repeated the Final models but used a different outcome. Instead of N (total number of consultations per month) we used N_unique (the total number unique patients per month, i.e., if a patient visited the GP two times in one month this was counted as 1)  Subgroup analysis: We repeated Model_1 in a) subgroup of only girls aged 13-17 and b) subgroup of only young women aged 18-24. |

*-*

eTable 3. Model 1-3 complete output

| **Variables** | **Model 1: Number of psychosocial contacts based on data up to February 2020** | | **Model 2: Number of non-psychosocial contact based on data up to February 2020** | | **Model 3: Number of psychosocial contacts based on data up to December 2021 with the variables ‘COVID-19 infection rate’ and ‘school closure’** | |
| --- | --- | --- | --- | --- | --- | --- |
|  | **Estimate of relative rate (RR) (95% CI)** | ***P* value** | **Estimate of relative rate (RR) (95% CI)** | ***P* value** | **Estimate of relative rate (RR)**  **(95% CI)** | ***P* value** |
| Trend (RR per subsequent month) | 1.009 (1.008-1.011) | *P* < 0.001 | 1.004 (1.003-1.005) | *P* < 0.001 | 1.010 (1.008-1.011) | *P* < 0.001 |
| February | 0.942 (0.862-1.029) | *P* = 0.185 | 0.981 (0.922-1.044) | *P* = 0.55 | 0.948 (0.866-1.038) | *P* = 0.248 |
| March | 1.080 (0.983-1.029) | *P* = 0.107 | 1.058 (0.99-1.131) | *P* = 0.094 | 1.066 (0.974-1.166) | *P* = 0.163 |
| April | 0.932 (0.848-1.024) | *P* = 0.142 | 0.962 (0.901-1.028) | *P* = 0.257 | 0.902 (0.824-0.987) | *P* = 0.024 |
| May | 0.907 (0.826-0.997) | *P* = 0.043 | 0.984 (0.921-1.052) | *P* = 0.641 | 0.875 (0.800-0.958) | *P* = 0.004 |
| June | 0.949 (0.863-1.042) | *P* = 0.273 | 0.988 (0.925-1.056) | *P* = 0.72 | 0.966 (0.882-1.057) | *P* = 0.449 |
| July | 0.795 (0.723-0.874) | *P* < 0.001 | 0.865 (0.809-0.924) | *P* = <0.001 | 0.799 (0.729-0.875) | *P* < 0.001 |
| August | 0.710 (0.646-0.781) | *P* < 0.001 | 0.816 (0.764-0.872) | *P* = <0.001 | 0.687 (0.627-0.753) | *P* < 0.001 |
| September | 0.877 (0.798-0.963) | *P* = 0.006 | 0.932 (0.873-0.996) | *P* = 0.038 | 0.882 (0.805-0.967) | *P* = 0.007 |
| October | 0.930 (0.846-1.021) | *P* = 0.129 | 0.952 (0.891-1.017) | *P* = 0.143 | 0.907 (0.828-0.994) | *P* = 0.037 |
| November | 1.020 (0.928-1.120) | *P* = 0.684 | 0.987 (0.924-1.055) | *P* = 0.702 | 0.975 (0.888-1.071) | *P* = 0.604 |
| December | 0.864 (0.786-0.949) | *P* = 0.002 | 0.931 (0.872-0.995) | *P* = 0.036 | 0.878 (0.800-0.963) | *P* = 0.005 |
| School closure (scale 0 to 1) | Not applicable | Not applicable | Not applicable | Not applicable | 0.786 (0.706-0.875) | *P* < 0.001 |
| New COVID-19 infections (number/1000 inhabitants) | Not applicable | Not applicable | Not applicable | Not applicable | 1.010 (1.002-1.018) | *P* = 0.010 |

*Model 1 <- glm.nb(n ~ trend + month + offset(log(patient months)), data = Psychosocial consultations, Jan 2016 – Feb 2020)*

*Model 2 <- glm.nb(n ~ trend + month + offset(log(patient months)), data = Non- Psychosocial consultations, Jan 2016 – Feb 2020)*

*Model 3 <- glm.nb(n ~ trend + month + School closure + New COVID-19 infections + offset(log(patient months)), data = Psychosocial consultations ,Jan 2016 – Dec 2021)*

eFigure 1a. Observed consultation rates for psychosocial problems over time (Males)
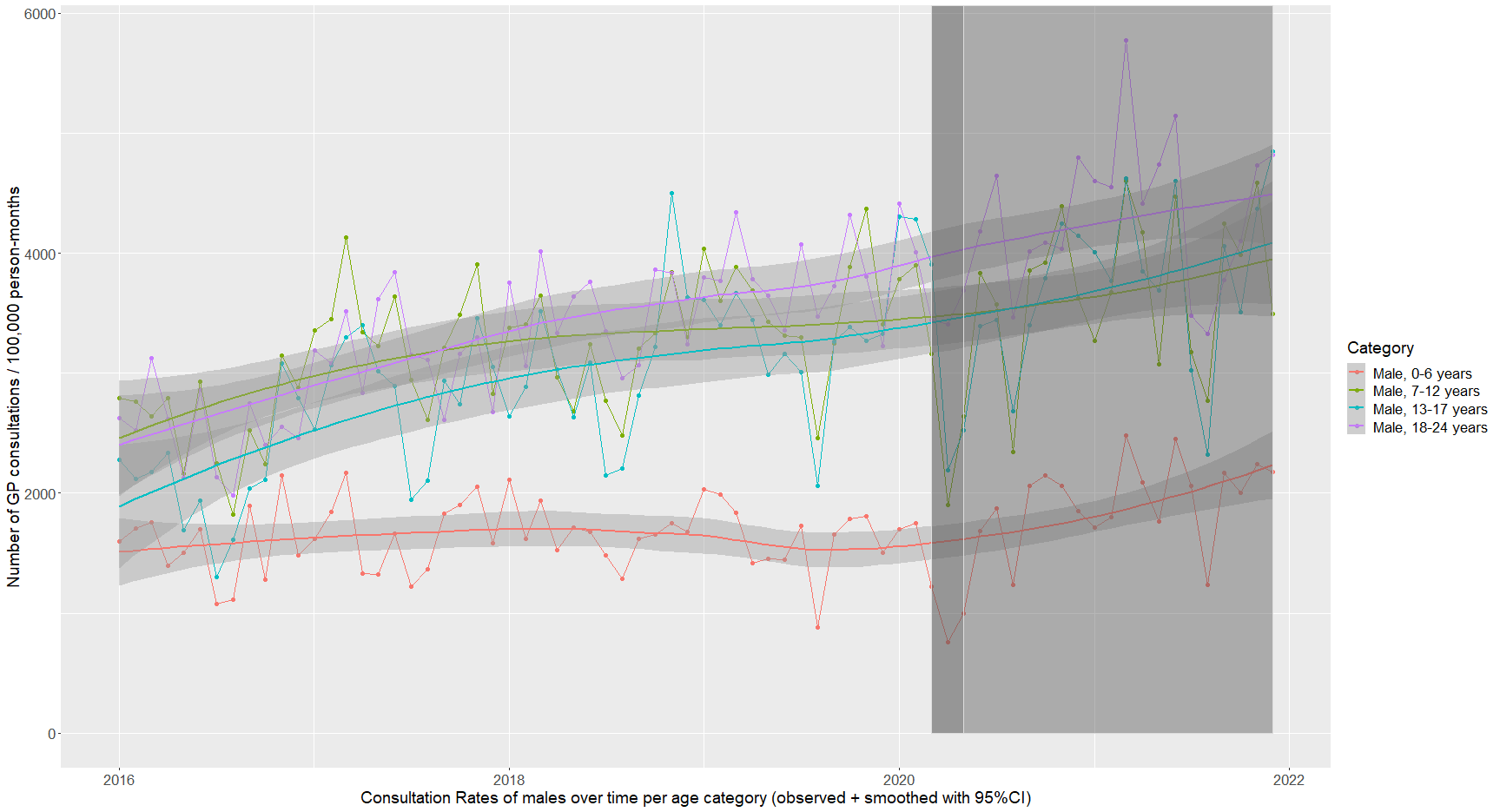


*Monthly observed consultation rates per age group and sex, including smoothed line. The shaded area indicates the Covid-19 pandemic, the dark shaded area covers the first Covid-19 wave.*

eFigure 1b. Observed consultation rates for psychosocial problems over time (Females)

*
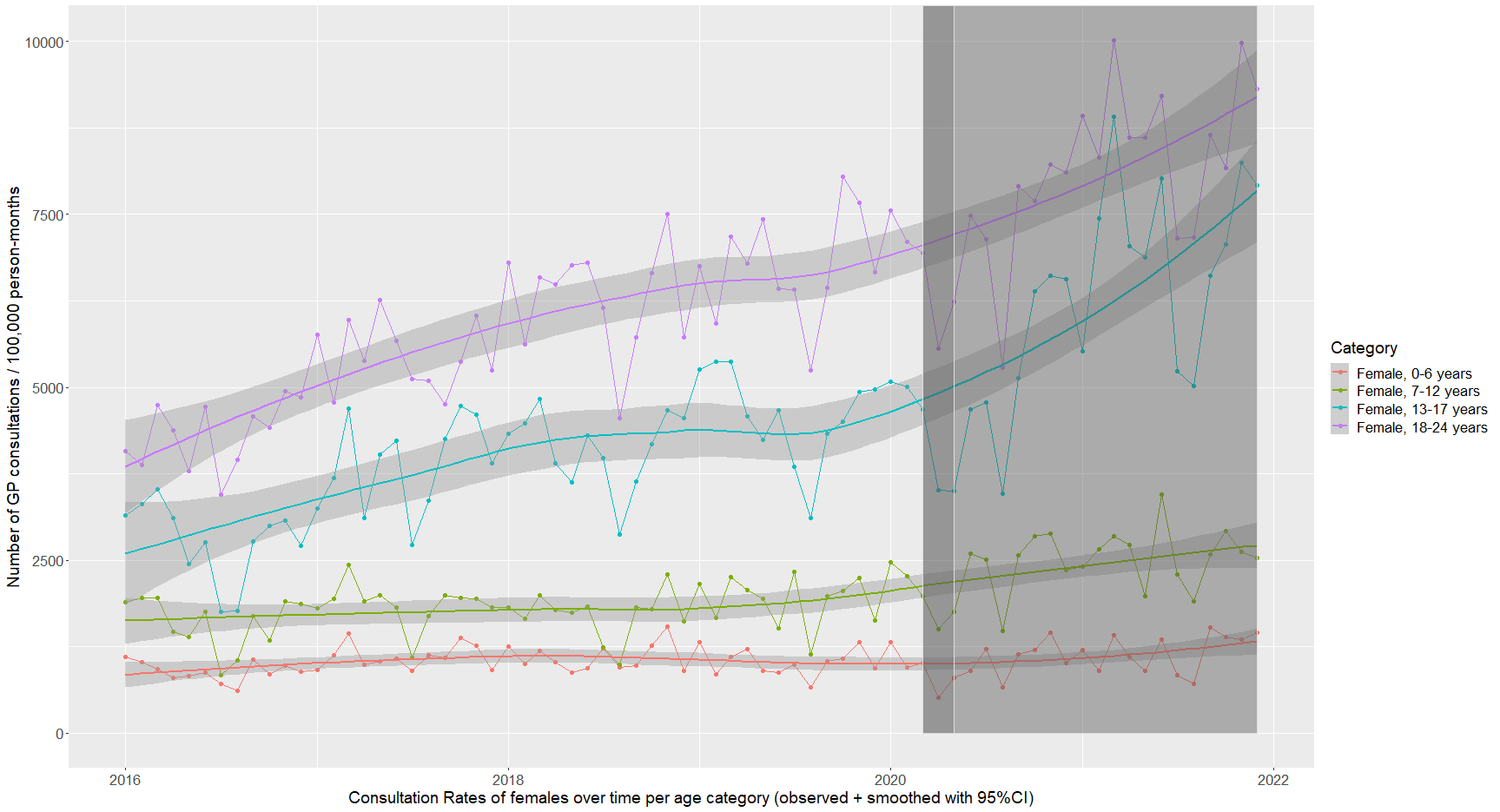
*

*Monthly observed consultation rates per age group and sex, including smoothed line. The shaded area indicates the Covid-19 pandemic, the dark shaded area covers the first Covid-19 wave.*

eFigure 2. Monthly Consultation rates for non-psychosocial problems patients per age category and sex

*
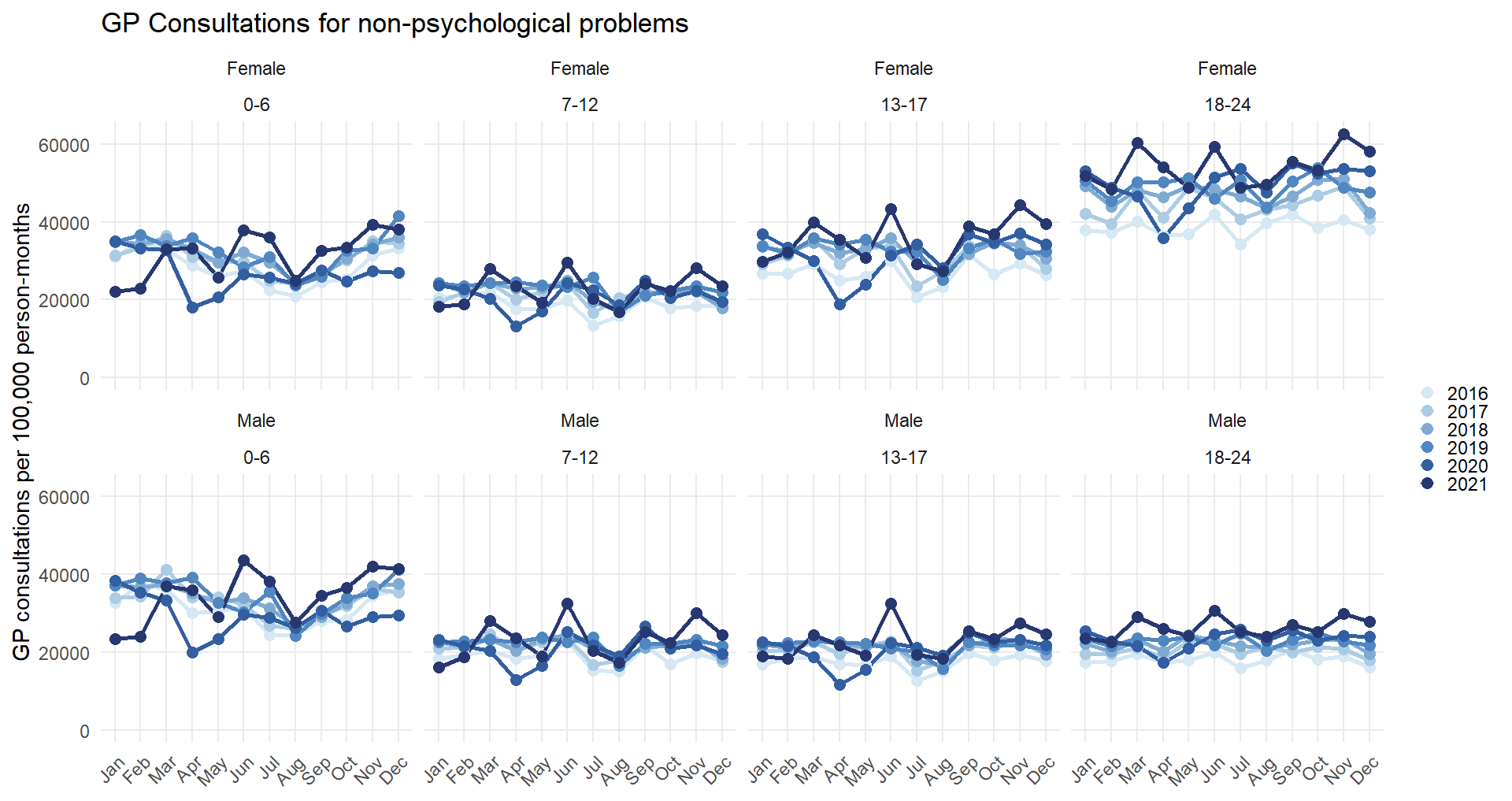
*

eTable 4. Observed number of consultations for psychosocial problems, compared with expected numbers of consultations based on Model 1 and 3.

| **Standardised for 100.000 children** | **Observed number of psychosocial consultations** | **Model 1: Expected number of psychosocial consultations based on data up to February 2020** | **Model 3: Expected number of psychosocial consultations based on data up to December 2021 with the variables ‘COVID-19 infection rate’ and ‘school closure’ set to 0*** |
| --- | --- | --- | --- |
| First COVID-19 lockdown:  March 2020 – May 2020 | 7382 | 10262 (95%CI 9459 -11134) | 10162 (95%CI 9413-10972) |
| Complete COVID-19 period:  March 2020 – December 2021 | 82941 | 87637 (95CI% 80398-94876) | 88324 (95CI% 81485-95163) |

* To validate our estimates, we did a second approach to estimate consultation rates assuming the absence of the COVID-19 pandemic, by setting ‘school-closure index’ and ‘COVID-19 infection rate’ to 0. As can be seen from the table, this gave very comparable results.

eTable 5. Regression table of Model 4, based on consultation rate per age-category and sex, including interactions

| **Variable** | **Estimate of relative rate (RR) (95% CI)** | ***P* value** |
| --- | --- | --- |
| Time trend | 1.009 (1.007-1.010) | *P* < 0.001 |
| February | 0.957 (0.855-1.071) | *P* = 0.446 |
| March | 1.067 (0.954-1.194) | *P* = 0.257 |
| April | 0.884 (0.789-0.990) | *P* = 0.033 |
| May | 0.85 (0.759-0.952) | *P* = 0.005 |
| June | 0.962 (0.859-1.078) | *P* = 0.505 |
| July | 0.793 (0.708-0.889) | *P* < 0.001 |
| August | 0.669 (0.597-0.750) | *P* < 0.001 |
| September | 0.902 (0.805-1.012) | *P* = 0.078 |
| October | 0.912 (0.813-1.023) | *P* = 0.115 |
| November | 0.987 (0.878-1.110) | *P* = 0.826 |
| December | 0.873 (0.778-0.981) | *P* = 0.022 |
| Age 7-12 | 1.862 (1.725-2.010) | *P* < 0.001 |
| Age 13-17 | 2.513 (2.329-2.712) | *P* < 0.001 |
| Age 18-24 | 3.361 (3.116-3.626) | *P* < 0.001 |
| New COVID-19 cases | 1.013 (1.003-1.023) | *P* = 0.009 |
| **Interaction** | | |
| School closure in:  Age 0-6  Age 7-12  Age 13-17  Age 18-24 | 0.563 (0.459-0.692)  0.693 (0.565-0.848)  0.951 (0.777-1.163)  0.891 (0.729-1.089 | *P* < 0.001^c^ |

*Model 4 <- glm.nb(n ~ trend + month + age category + sex + school closure + COVID-19 infections + (age category : school closure) + offset(log(patient months)), data = Psychosocial consultations, Jan 2016 – Dec 2021)*

eFigure 3. Monthly unique patients consulting general practice for psychosocial problems per age category and sex


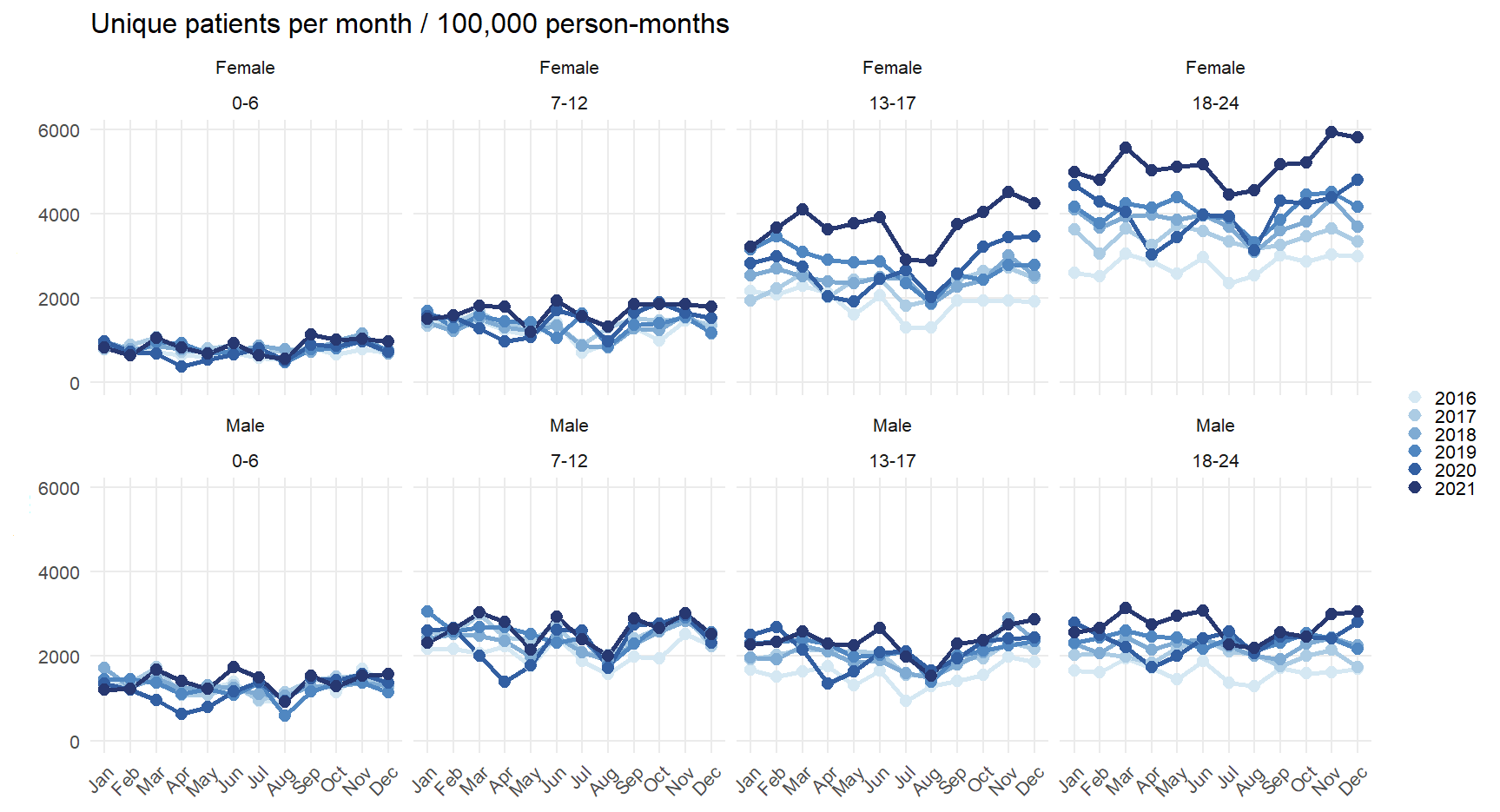


eFigure 4. Observed monthly unique patients consulting general practice for psychosocial problems rates versus expected based on data until February 2020*
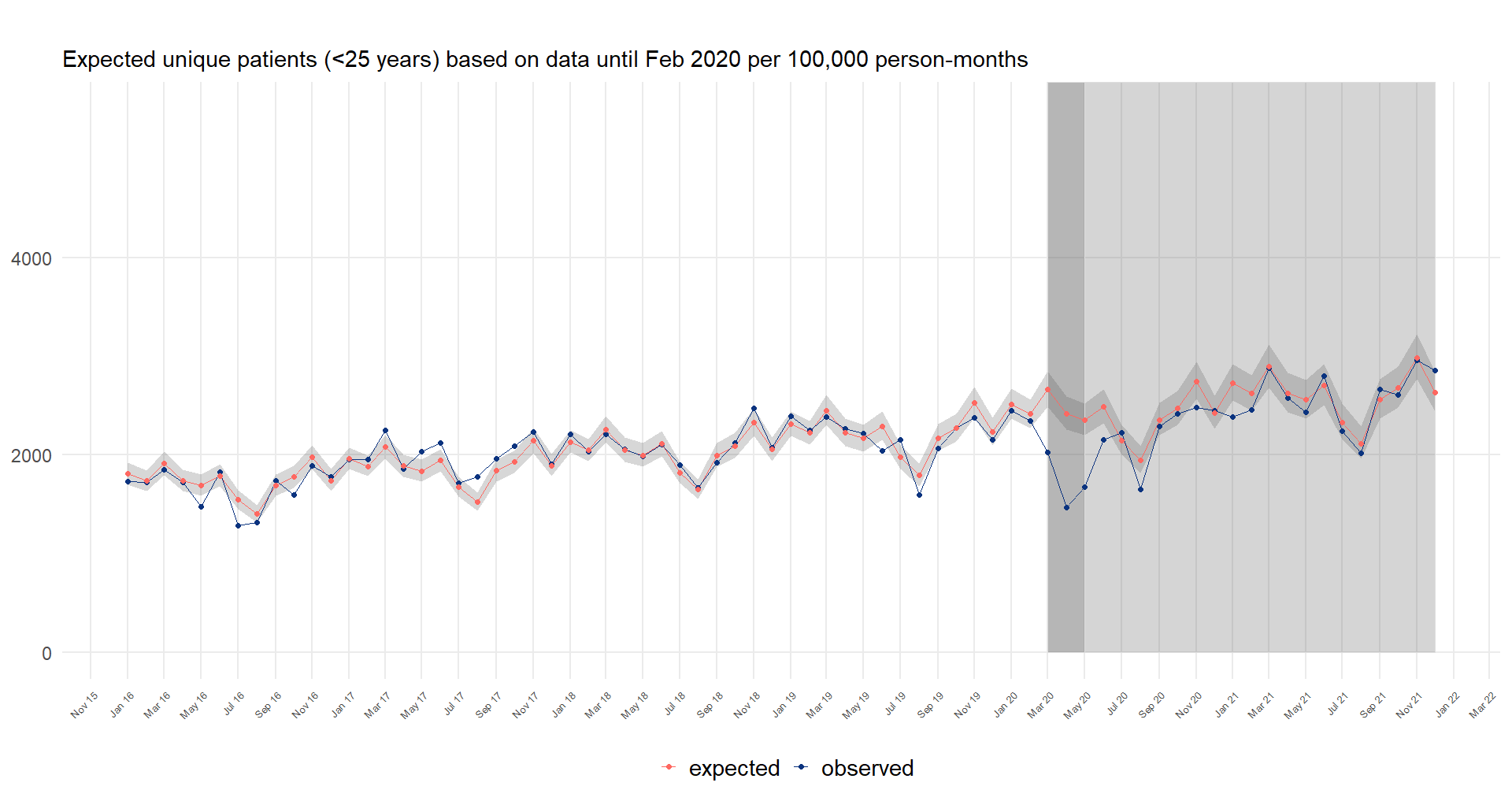
*

eFigure 5a. Subgroup analysis girls aged 13-17. Observed monthly consultation rates versus expected rates based on data until February 2020


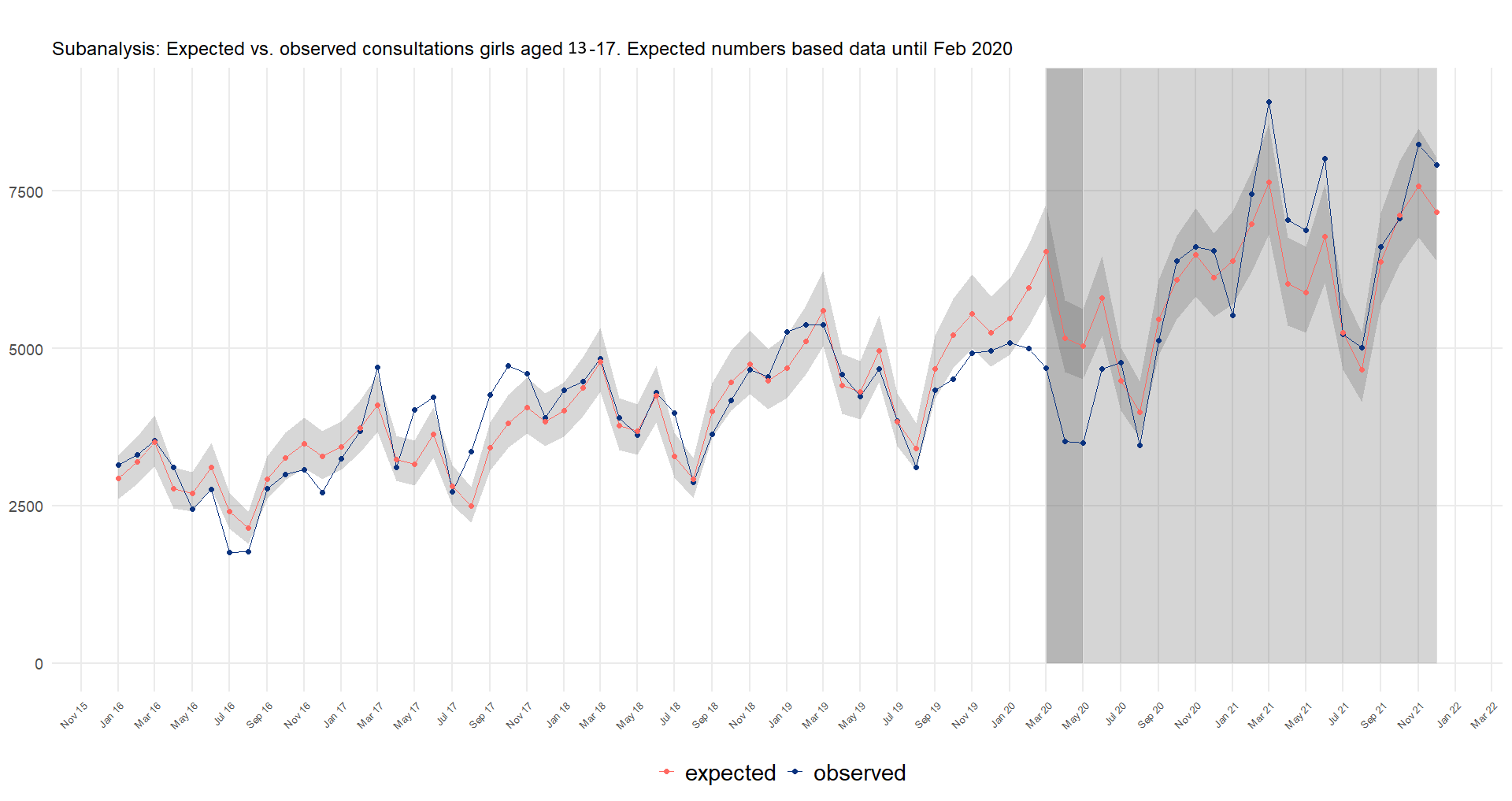


eFigure 5b. Subgroup analysis young women aged 18-24, observed monthly consultation rates versus expected rates based on data until February 2020


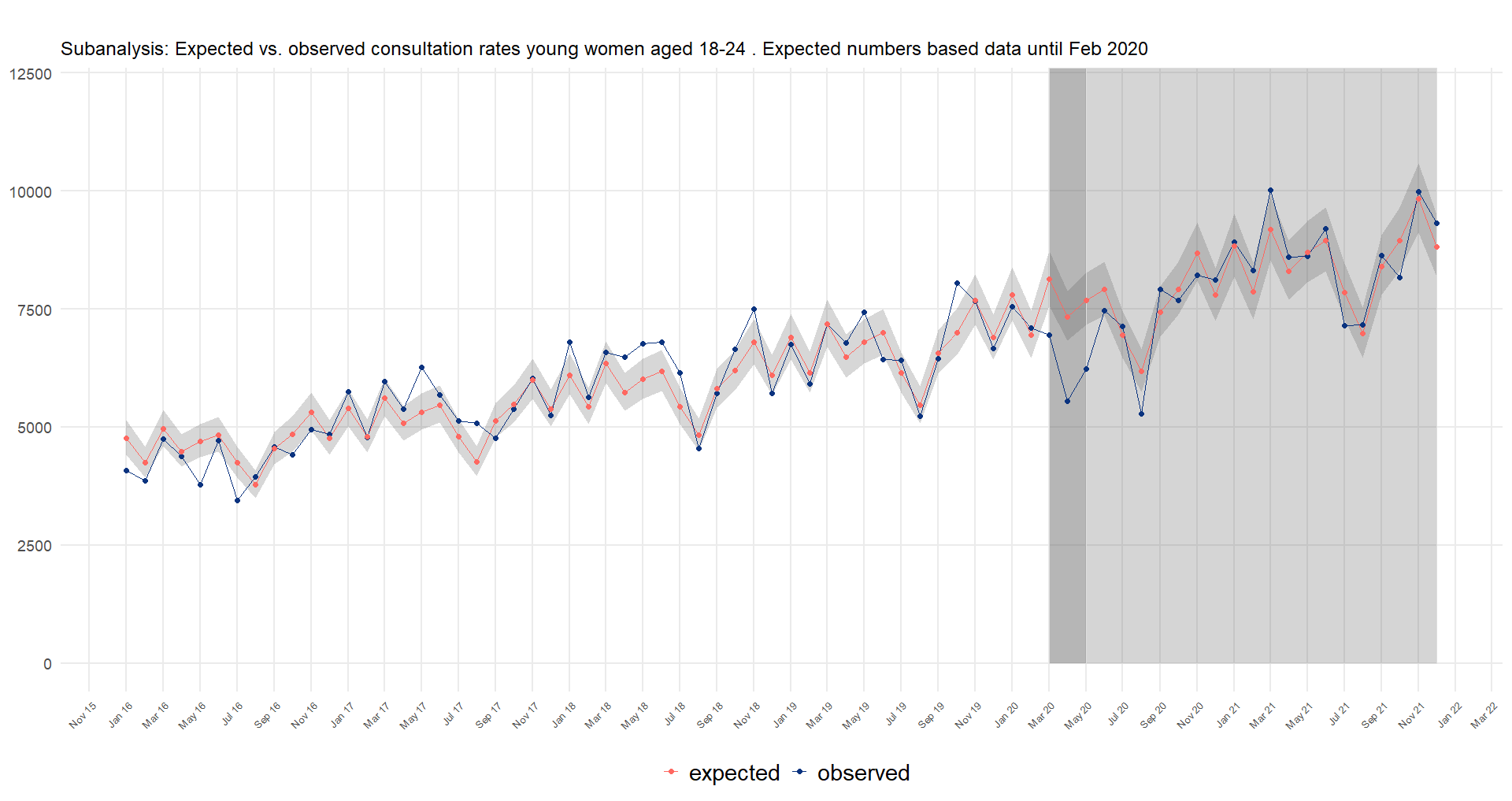


eTable 6: Sensitivity analysis unique patients

| **Variable** | **Model of monthly unique patients based on data up to February 2020** | | **Model of monthly unique patients based on data up to December 2021 with COVID-19 indices** | |
| --- | --- | --- | --- | --- |
|  | Estimate of relative rate (RR) (95% CI) | Pr(>\|z\|) | Estimate of relative rate (RR) (95% CI) | *P-v*alue |
| Trend (RR per subsequent month) | 1.007 (1.006-1.008) | *P* < 0.001 | 1.007 (1.005-1.01) | *P* = <0.001 |
| February | 0.954 (0.885-1.028) | *P* = 0.214 | 0.962 (0.802-1.155) | *P* = 0.681 |
| March | 1.045 (0.965-1.13) | *P* = 0.28 | 1.034 (0.862-1.241) | *P* = 0.718 |
| April | 0.942 (0.87-1.019) | *P* = 0.137 | 0.91 (0.758-1.093) | *P* = 0.314 |
| May | 0.911 (0.842-0.986) | *P* = 0.022 | 0.876 (0.73-1.051) | *P* = 0.154 |
| June | 0.956 (0.884-1.035) | *P* = 0.270 | 0.962 (0.801-1.156) | *P* = 0.682 |
| July | 0.819 (0.757-0.887) | *P* = <0.001 | 0.815 (0.678-0.98) | *P* = 0.029 |
| August | 0.738 (0.682-0.8) | *P* = <0.001 | 0.714 (0.594-0.859) | *P* = <0.001 |
| September | 0.887 (0.819-0.96) | *P* = 0.003 | 0.895 (0.744-1.077) | *P* = 0.239 |
| October | 0.923 (0.853-0.999) | *P* = 0.048 | 0.903 (0.75-1.087) | *P* = 0.283 |
| November | 1.020 (0.943-1.104) | *P* = 0.622 | 0.967 (0.8-1.169) | *P* = 0.730 |
| December | 0.894 (0.826-0.968) | *P* = 0.006 | 0.9 (0.747-1.086) | *P* = 0.272 |
| School Closure | Not applicable | Not applicable | 0.728 (0.585-0.905) | *P* = 0.004 |
| New COVID-19 infections (number/1000 inhabitants) | Not applicable | Not applicable | 1.015 (0.999-1.031) | *P* = 0.072 |

*Model <- glm.nb(n ~ trend + month + offset(log(patient months)), data = Unique patients, Jan 2016 – Feb 2020)*

*Model <- glm.nb(n ~ trend + month + School closure + New COVID-19 infections + offset(log(patient months)), data = Unique patients, Jan 2016 – Dec 2021)*

eTable 7. Sensitivity analysis unique patients with interaction between age category using data up to December 2021

| **Variable** | **Relative rate (95%CI)** | ***P* value** |
| --- | --- | --- |
| Trend (RR per subsequent month) | 1.006 (1.005-1.008) | *P* < 0.001 |
| February | 0.959 (0.862-1.067) | *P* = 0.443 |
| March | 1.035 (0.931-1.152) | *P* = 0.522 |
| April | 0.897 (0.806-0.998) | *P* = 0.046 |
| May | 0.859 (0.772-0.956) | *P* = 0.005 |
| June | 0.959 (0.861-1.067) | *P* = 0.443 |
| July | 0.815 (0.732-0.907) | *P* < 0.001 |
| August | 0.703 (0.63-0.783) | *P* < 0.001 |
| September | 0.908 (0.815-1.011) | *P* = 0.079 |
| October | 0.908 (0.815-1.012) | *P* = 0.082 |
| November | 0.977 (0.874-1.091) | *P* = 0.677 |
| December | 0.894 (0.801-0.997) | *P* = 0.044 |
| Male gender | 1.048 (1.003-1.094) | *P* = 0.036 |
| Age 7-12 | 1.773 (1.65-1.907) | *P* < 0.001 |
| Age 13-17 | 2.083 (1.937-2.239) | *P* < 0.001 |
| Age 18-24 | 2.733 (2.544-2.936) | *P* < 0.001 |
| New COVID-19 infections (number/1000 inhabitants) | 1.016 (1.006-1.025) | *P* = 0.001 |
| School closure * Age 0-6 (Ref category)  School closure * Age 7-12 (compared to risk of Ref category)  School closure * Age 13-17 (compared to risk of Ref category)  School closure * Age 18-24  (compared to risk of Ref category) | 0.55 (0.452-0.669)  1.183 (0.934-1.499)  1.538 (1.215-1.948)  1.577 (1.248-1.994) | *P* < 0.001 |

*Model <- glm.nb(n ~ trend + month + sex + age category + COVID-19 infections + school closure + (school closure : age category) + offset(log(totals)) , data = Unique patients, Jan 2016 – Dec 2021)*

eTable 8. Subgroup analysis, observed number of consultations for psychosocial problems, compared with expected numbers of consultations based on data until February 2020

|  | **Girls aged 13-17** | | **Young women aged 18-24** | |
| --- | --- | --- | --- | --- |
|  | Observed number of consultations | Expected number of consultations (95%CI) | Observed number of consultations) | Expected number of consultations (95%CI) |
| **First COVID-19 lockdown:**  **March 2020 – May 2020** | 1007 | 1441 (1292-1608) | 2319 | 2867 (2669-3080) |
| **Complete COVID-19 period:**  **March 2020 – December 2021** | 13089 | 12974 (11590-14358) | 24160 | 24645 (22892-26398) |
